# The Quality of Evidence of and Engagement With Video Medical Claims

**DOI:** 10.1001/jamanetworkopen.2025.52106

**Published:** 2026-01-16

**Authors:** EunKyo Kang, HyeWon Lee, Juyoung Choi, HyoRim Ju

**Affiliations:** 1 National Cancer Control Institute, National Cancer Center, Goyang, Korea; 2 National Cancer Center Graduate School of Cancer Science and Policy, National Cancer Center, Goyang, Korea; 3 Department of Family Medicine, National Cancer Center, Goyang, Korea; 4 Department of Family Medicine, Dankook University Hospital, Cheonan, Korea; 5 Department of Family Medicine, Seoul National University College of Medicine, Seoul, Korea

## Abstract

**Importance:**

The unexplored quality of evidence supporting online video claims by medical professionals creates a credibility-evidence gap that threatens the principles of evidence-based medicine.

**Objective:**

To systematically evaluate the evidence hierarchy supporting medical claims in health care professional–created online videos using a novel evidence classification framework.

**Design, Setting, and Participants:**

In this quality improvement study using a cross-sectional analysis, YouTube was searched using cancer- and diabetes-related terms. A total of 309 videos met the inclusion criteria. The video search, data extraction, and archiving were conducted between June 20 and 21, 2025, to create a static dataset. Videos were assessed using the newly developed Evidence-GRADE (E-GRADE [Grading of Recommendations Assessment, Development and Evaluation]) framework, categorizing evidence into 4 levels: grade A (high certainty from systematic reviews and/or guidelines), grade B (moderate certainty from randomized clinical trials, cohort studies, and high-quality observational studies with clear citations), grade C (low certainty from limited observational studies, physiological mechanisms, or case series without critical appraisal), and grade D (very low or no certainty from anecdotal evidence).

**Exposure:**

Videos that had a minimum of 10 000 views, were created by health care professionals, had a minimum duration of 1 minute, and contained specific health claims.

**Main Outcomes and Measures:**

Primary outcomes included the distribution of evidence grades (A-D) supporting medical claims. Secondary outcomes included correlations between evidence quality and engagement metrics (views and likes) and traditional quality scores (DISCERN, *JAMA* benchmark criteria, and Global Quality Scale).

**Results:**

Among the 309 videos included, which had a median of 164 454 (IQR, 58 909-477 075) views, most medical claims (193 [62.5%]) were supported by very low or no evidence (grade D), while only 61 claims (19.7%) were supported by high-quality evidence (grade A). Moderate (grade B) and low (grade C) evidence levels were found in 45 (14.6%) and 10 (3.2%) videos, respectively. The correlation with view counts was statistically significant for grade D videos, which were associated with a 34.6% higher view count (incidence rate ratio, 1.35; 95% CI, 1.00-1.81; *P* = .047) than grade A videos. Traditional quality tools showed only weak correlations (range of coefficients, 0.11-0.23) with evidence levels, thus failing to detect important qualitative differences.

**Conclusions and Relevance:**

In this quality improvement study, a substantial credibility-evidence gap was found in physician-generated video-sharing content, where medical authorities often legitimized claims lacking robust empirical support. These findings emphasize the need for evidence-based content guidelines and enhanced science communication training for health care professionals to maintain scientific integrity in digital health information.

## Key Points

**Question:**

Are medical claims in health care professional–created online videos supported by strong scientific evidence, and are evidence levels associated with engagement metrics or traditional video quality assessment tools?

**Findings:**

In this quality improvement study of 309 video claims, 62% of claims relied on very little or no evidence, while less than 20% were supported by high-quality evidence. Grade D videos (ie, those with very low or no certainty of evidence) were associated with a 35% higher view count than grade A videos (ie, those with very high certainty of evidence), and traditional quality tools showed weak correlations with evidence levels, thus failing to detect important qualitative differences.

**Meaning:**

These findings suggest that to maintain scientific integrity in digital health communication, evidence-based content standards and improved science communication training for health care professionals are needed.

## Teaser

This quality improvement study evaluates the hierarchical level of evidence supporting the claims presented in videos posted by health care professionals.

## Introduction

The contemporary health care landscape has transformed significantly in how patients access and consume medical information. YouTube (hereinafter referred to as the study platform) has emerged as a primary social media platform for seeking health information globally, particularly among younger populations, fundamentally altering the traditional patient journey.^1^ Patients actively use the platform to explore health conditions before clinical consultations, learn about their diseases, and even make decisions regarding specific medical services.^2^ This phenomenon marks a shift from medical professionals as knowledge gatekeepers to a digital space lacking traditional editorial gatekeeping, where anyone can produce and consume information.

Modern medical professionals are now content creators, expanding their influence beyond the clinic to educate and engage a wider audience.^3,4^ These physicians leverage video-sharing platforms to share expertise, fostering patient trust and improving treatment adherence.^5,6^ However, this new role presents profound ethical challenges, blurring the lines among professional decorum, public communication, and the boundaries of personal and professional life.^4,7,8^ While video-sharing platforms provide unprecedented access to information, the absence of peer review and regulatory oversight has allowed them to become saturated with misleading, inaccurate, and incomplete content.^9^ This “infodemic” makes it difficult for the public to separate scientific facts from unverified claims, which could lead to serious health risks for viewers.^10,11^

The severity of this problem is highlighted by a credibility paradox, wherein even content produced by medical professionals often exhibits disappointingly low information quality.^12^ This issue has worsened as lower-quality or overtly false information gains greater popularity, with multiple studies demonstrating that misinformation frequently achieves higher view counts, likes, and comments than evidence-based content from authoritative sources.^13,14^Recommendation algorithms may inadvertently prioritize sensationalized or simplified narratives over scientifically rigorous information.^11,15^

The study platform has implemented policies to combat medical misinformation, categorizing it into the areas of prevention and treatment, while removing content that promotes harmful treatments or contradicts guidance from health authorities such as the World Health Organization.^16^ The platform has also introduced authentication marks to identify trustworthy sources and features to label videos from licensed health care professionals.^17^ However, the effectiveness of these measures remains controversial, and the fundamental problem of assessing the level of evidence for claims made, even from seemingly trustworthy sources, remains largely unaddressed.

Recent studies have used various validated quality rating tools to assess medical information provided on video-sharing platforms, including the DISCERN score (a tool for evaluating the quality of consumer treatment choice information),^18^ *JAMA* benchmark criteria (4 criteria evaluating authorship, attribution, disclosure, and currency of information),^19^ and the Global Quality Scale score (GQS; evaluating educational value and flow of videos on a scale of 1 to 5).^20^ These studies consistently conclude that while physician-produced content tends to achieve higher overall quality scores than lay-produced content,^21^ the general information quality across the platform remains low, and engagement metrics prove to be unreliable proxies for information accuracy.^22^

This study addresses a critical gap overlooked by previous research. Previous studies have focused on measuring overall quality, reliability, and accuracy but have not systematically evaluated the hierarchical level of evidence supporting the claims presented in videos.^23,24^ For instance, a video may receive high scores on the DISCERN evaluation because the producer is a physician and the video has a clear purpose and high production quality, but its recommendations could rely only on personal experiences or anecdotes, which are at the lowest level of the evidence pyramid. This research moves beyond the question “Is the information presented well?” to a more fundamental issue: “How strong is the evidence supporting the claims themselves?” This approach is essential for analyzing the credibility-evidence gap, a phenomenon where physician authority acts as a halo effect, giving scientific legitimacy to claims that may not have strong empirical backing, even if they come from credentialed sources.^8^ This study proposes a new framework to address a critical threat to public scientific literacy and the principles of evidence-based medicine by analyzing and quantifying this gap using established evidence hierarchies.

## Methods

This quality improvement study was prepared in accordance with the Standards for Quality Improvement Reporting Excellence (SQUIRE 2.0) reporting guideline. The study was determined to be exempt from review and the need for informed consent by the National Cancer Center Institutional Review Board, as it involved publicly available data and did constitute human participant research.

### Video Collection and Selection

A comprehensive search strategy was developed to reflect actual user search behaviors by combining both lay terminology and medical terms. Two primary health conditions were selected as search topics: cancer and diabetes. The selection of cancer and diabetes as search topics was purposeful. These conditions were chosen due to their high global prevalence, substantial public interest, and the large volume of related content on social media. Together, they represent a broad spectrum of health information, from lifestyle management and prevention (common in diabetes content) to complex treatment and prognostic discussions (common in cancer content), allowing for a more comprehensive analysis of medical claims online. The search was conducted using the study platform’s native search function with cache-cleared browsers to minimize personalization algorithm effects. A comprehensive list of all search terms used in both languages is provided in eAppendix 1 in Supplement 1. The video search was conducted on June 20 and 21, 2025, using Korean-language search terms. The analysis was not limited by the creator’s country of origin; any Korean- or English-language video meeting the criteria was included to reflect the global nature of the platform.

Videos were included if they met the following criteria: (1) minimum view count of 10 000 to ensure adequate public exposure; (2) content created by a medical professional (physician, dentist, doctor of traditional Korean medicine, pharmacist, nurse, herbal pharmacist, and others); (3) minimum duration of 1 minute to exclude the study platform’s short video format; (4) contained specific, actionable health claims related to treatment, diagnosis, or prevention; and (5) available in Korean or English. The scope of topics was intentionally broad; videos concerning any aspect of these conditions (eg, prevention, diagnosis, treatment, specific subtypes such as type 1 or 2 diabetes, or any cancer type) were eligible for inclusion. Videos were excluded if they (1) were duplicates or lacked audio content; (2) were created by non–health care professionals; (3) contained purely promotional content without medical claims or were news broadcasts; or (4) were targeted at other health care professionals (including trainees) rather than the public. This final exclusion was based on the videos’ distinct communicative purpose, use of specialized language, and assumed expert knowledge, which fell outside the scope of public-facing health communication.

The systematic process of video identification, screening, and inclusion is detailed in the flow diagram provided in eFigure 1 in Supplement 1. Our initial search yielded 1582 videos. After the removal of 207 duplicates, 1375 unique videos were screened by title and duration, leading to the exclusion of 822 videos (612 for being shorter than 1 minute; 210 for irrelevant contents). The remaining 553 videos underwent full eligibility assessment, from which a further 244 were excluded for not meeting the inclusion criteria. This resulted in a final sample of 309 videos included in the analysis. Of these, 164 videos (53.1%) addressed cancer-related topics and 145 (46.9%) focused on diabetes management and treatment.

The study did not apply a specific publication date range for video inclusion. The earliest video in our final sample was published on December 21, 2018. To manage the ephemeral nature of online content, our primary data preservation method was to systematically archive the URL and all metadata for each included video at the time of collection.

### Evidence Classification Framework

A novel evidence classification system, termed Evidence-GRADE (E-GRADE), was developed based on the established GRADE (Grading of Recommendations Assessment, Development and Evaluation) methodology. This framework categorizes the evidence level supporting medical claims presented in the videos into 4 distinct grades:

- Grade A (high certainty): Claims are explicitly supported by systematic reviews, meta-analyses, or established clinical practice guidelines from major health organizations (defined as national or international, government-recognized, or large nonprofit professional bodies, eg, the World Health Organization, the American Dental Association).
- Grade B (moderate certainty): Claims are backed by specific randomized clinical trials or high-quality observational studies with clear citations (operationalized as large-scale prospective cohorts or well-designed case-control studies).
- Grade C (low certainty): Claims are supported by limited observational studies (eg, smaller retrospective studies, case series), physiological mechanisms, or case series without critical appraisal.
- Grade D (very low or no certainty): Claims are based solely on anecdotal evidence, personal experience, or unsupported assertions.

### Data Extraction and Coding

Two independent researchers (E.K.K. and H.W.L.) conducted standardized data extraction using a structured coding sheet (eAppendix 2 in Supplement 1). Video characteristics recorded included creator characteristics, video metrics (views, likes, and comments), and content features. Individual medical claims within each video were identified and classified according to the E-GRADE framework. The assessment of each claim followed a systematic protocol. First, the 2 independent researchers identified the primary actionable health claim within each video. They then examined the video and its description for any cited evidence. When a specific citation was present, the researchers retrieved the full source and performed a claim-to-evidence matching process to verify that the source directly supported the claim. The E-GRADE was then assigned based on the quality of this matched source. If evidence was absent or cited vaguely (eg, a general statement such as “studies show”), the researchers conducted a systematic literature search using PubMed, the Cochrane Library, and major clinical guideline databases to find the highest level of current evidence supporting the claim. This evidence was then mapped to the E-GRADE framework. For instance, a claim was rated grade D if it was based solely on an anecdote or if our literature search found no supporting evidence from observational studies or higher. All coding was performed independently, and disagreements were resolved through discussion and adjudication by a third senior reviewer (H.R.J.) to ensure interrater reliability. Interrater reliability was assessed using Cohen κ statistics, which demonstrated a high degree of agreement between the 2 independent coders across all instruments (Cohen κ range, 0.97-0.98), with all 95% CIs indicating high precision (95% CI range, 0.95-0.10). Disagreements were resolved through discussion and adjudication by the third senior reviewer. Furthermore, videos were evaluated to enable comparative analysis using established quality assessment tools. For our comparative analysis, we selected 3 of the most widely used instruments: DISCERN, a 16-item tool designed to help consumers assess the quality of written health information on treatment choices, focusing on reliability and detail (scores range from 16-80, with higher scores indicating higher quality); the *JAMA* Benchmark Criteria (hereinafter referred to as *JAMA*), which assesses content based on 4 key indicators of trustworthiness (authorship, attribution, disclosure, and currency; higher scores indicating better trustworthiness); and the GQS, a 5-point scale that provides an overall assessment of a video’s educational value and quality of information flow (higher scores indicating better value and flow).

### Statistical Analysis

All statistical analyses were conducted using Python, version 3.11.13 (Python Software Foundation) within the Google Colaboratory Environment. Descriptive statistics were used to summarize video characteristics, expressed as means (SDs), medians (IQRs), and frequencies with percentages, as appropriate. For the comparative analysis, videos were first stratified into 4 groups based on their E-GRADE level (A, B, C, or D). The Kruskal-Wallis test was then used to compare the distributions of continuous variables (GQS, *JAMA*, DISCERN scores, and engagement metrics) across these 4 evidence-based groups, with post hoc pairwise comparisons performed when relevant. A multivariate negative binomial regression model was used to identify factors independently associated with video view counts, as this model is appropriate for overdispersed count data. The model included E-GRADE as the primary independent variable of interest, with disease (topic), log(channel size), months since upload, and log(video length) included as covariates. Results are presented as incidence rate ratios (IRRs) with 95% CIs. To further evaluate the strength and direction of the association between the E-GRADE levels and other key variables, Spearman rank correlation coefficients were calculated. Multiple linear regression analysis was applied to identify factors independently associated with video view counts, with results expressed as coefficients (β) with SEs and *P* values. A 2-sided *P* < .05 was considered statistically significant.

## Results

### Video Characteristics and Quality Assessment

A total of 309 health-related videos were analyzed (Table 1). Most of these videos were produced by physicians (n = 233), followed by doctors of traditional Korean medicine (n = 44), pharmacists (n = 22), nurses (n = 6), traditional pharmacists (n = 2), and dentists (n = 2). The mean (SD) video length was 19.0 (17.4) minutes, and the median time since upload was 8.1 (IQR, 4.2-20.7) months. In terms of viewer engagement, the median number of views was 164 454 (IQR, 58 909-477 075) and the median number of likes was 3373 (IQR, 1103-9286). Additionally, quality assessment revealed a mean (SD) GQS score of 3.2 (0.9), a mean (SD) *JAMA* score of 2.5 (1.0), and a mean (SD) DISCERN score of 51.5 (14.5).

**Table 1.** Video Characteristics and Quality Assessment

Characteristic	Values
Uploader classification, No. (%)	
Physician	233 (75.4)
Doctor of traditional Korean medicine	44 (14.2)
Pharmacist	22 (7.1)
Nurse	6 (1.9)
Korean pharmacist	2 (0.6)
Dentist	2 (0.6)
Video characteristics	
No. of videos	309
No. of unique creators	106
Video length, mean (SD), min	19.0 (17.4)
Elapsed time since upload, median (IQR), mo	8.1 (4.2-20.7)
Engagement metrics	
No. of views, median (IQR)	164 454 (58 909-477 075)
No. of likes, median (IQR)	3373 (1103-9286)
Quality assessment	
GQS score, mean (SD)^a^	3.2 (0.9)
*JAMA* benchmark criteria score, mean (SD)^b^	2.5 (1.0)
DISCERN score, mean (SD)^c^	51.5 (14.5)

Abbreviation: GQS, Global Quality Scale.

^a^ Scores range from 1 to 5, with higher scores indicating higher global quality.

^b^ Scores range from 1 to 4, with higher scores indicating better trustworthiness.

^c^ Scores range from 6 to 80, with higher scores indicating higher quality of information.

### Distribution of Evidence Levels for Video Claims

Among the claims in medical information videos (eFigure 2 in Supplement 1), 193 (62.5%) were classified as having very low or no evidence (grade D). Claims supported by high-level evidence (grade A) accounted for only 61 (19.7%), while moderate (grade B) and low (grade C) levels of evidence were observed in only 45 (14.6%) and 10 (3.2%) cases, respectively.

### Association Between E-GRADE and Video Engagement Metrics

**Figure 1.**
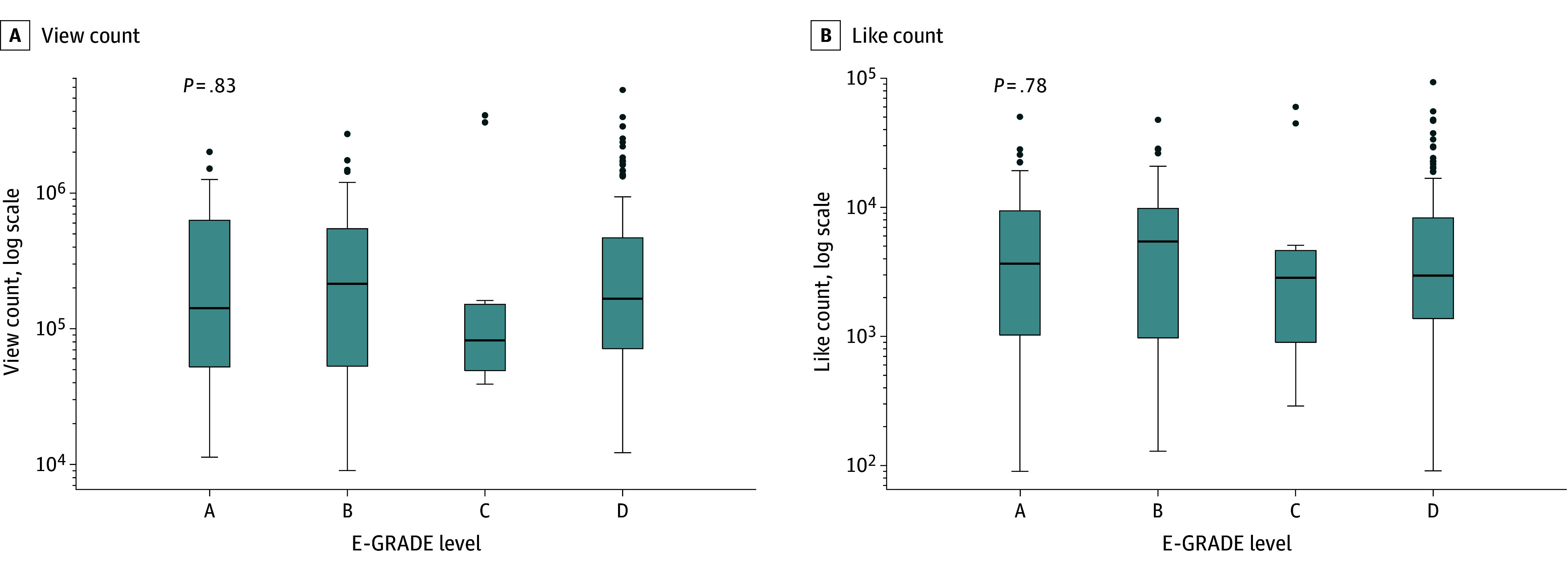
Engagement Metrics by Evidence-GRADE (E-GRADE [Grading of Recommendations Assessment, Development and Evaluation]) Level Boxes indicate the interquartile range; data points, outliers; error bars, 1.5 times the interquartile range; and horizontal bars, medians.

Analysis of video engagement metrics according to E-GRADE levels revealed no statistically significant differences in either view counts or like counts among the 4 groups. As shown in Figure 1, the median view counts and like counts, both presented on a logarithmic scale, were similar across E-GRADE groups (view count: 5.15 vs 5.33 vs 4.91 vs 5.22 [*P* = .83]; like count: 3.56 vs 3.74 vs 3.45 vs 3.47 [*P* = .78]). The results show that evidence quality (E-GRADE) does not correlate with user engagement, as indicated by view counts or likes.

Analysis of engagement efficiency using the Kruskal-Wallis test, defined as number of likes per 1000 views, revealed no significant differences among the E-GRADE groups (19.55 vs 18.33 vs 18.57 vs 17.29; *P* = .27). The median number of likes per 1000 views was generally similar across groups A, B, C, and D, and there was substantial overlap in their distributions (eFigure 3 in Supplement 1). The findings indicate that a video’s evidence grade does not seem to affect how efficiently viewers show positive engagement.

### Comparison of Quality Scores According to E-GRADE Levels

The quality assessment of the 4 groups (A, B, C, and D) was conducted using the GQS, *JAMA*, and DISCERN scoring systems (eTable in Supplement 1). The mean (SD) GQS scores were highest in group B (3.62 [1.01]), followed by group A (3.51 [0.77]), group D (3.06 [0.88]), and group C (2.90 [0.74]), with statistically significant differences among the groups (*P* < .001). *JAMA* scores did not differ significantly between groups, and mean (SD) DISCERN scores were significantly higher in groups A (55.44 [11.65]) and B (56.16 [13.93]) compared with groups C (45.10 [15.25]) and D (49.50 [14.91]) (*P* = .003).

**Figure 2.**
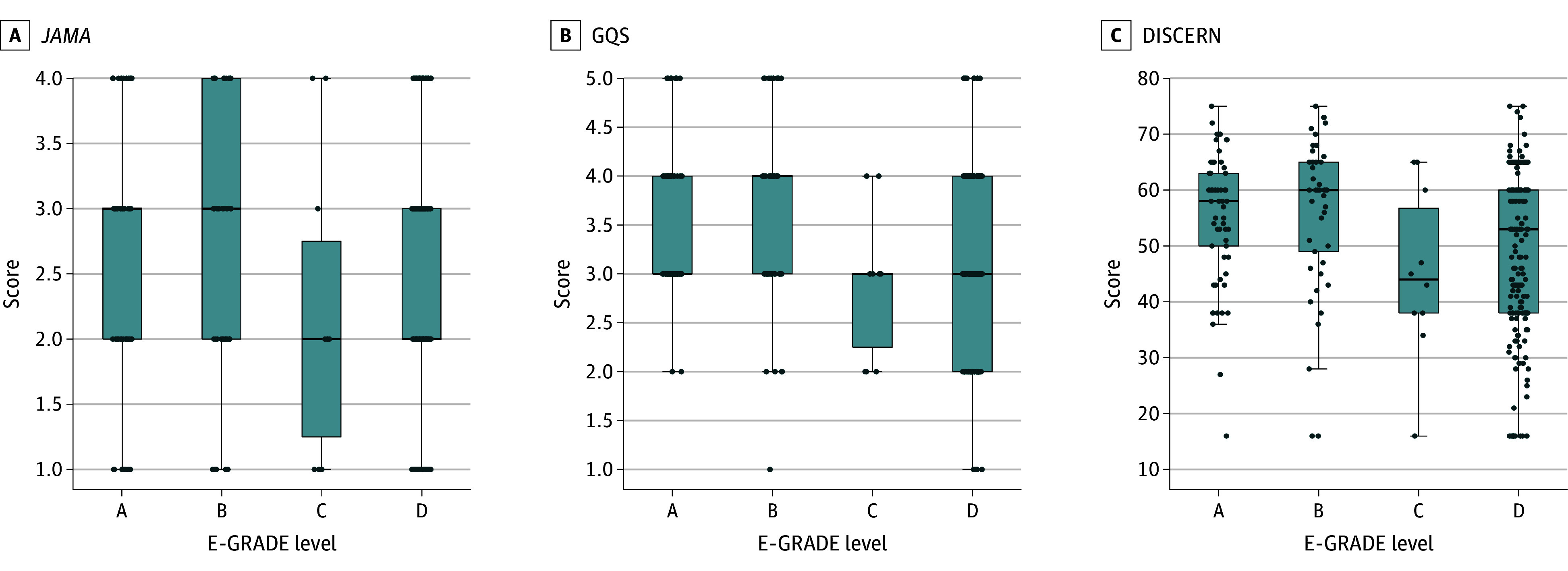
Distribution of Quality Scores by Evidence-GRADE (E-GRADE [Grading of Recommendations Assessment, Development and Evaluation]) Level DISCERN scores range from 16 to 80, with higher scores indicating higher quality; Global Quality Scale (GQS) scores, from 1 to 5, with higher scores indicating better value and flow; and *JAMA* benchmark criteria (*JAMA*), 1 to 4, with higher scores indicating better trustworthiness. Boxes indicate the interquartile range; data points, individual scores; error bars, 1.5 times the interquartile range; and horizontal bars, medians.

Figure 2 presents the distribution of 3 quality scores—*JAMA*, GQS, and DISCERN—across different E-GRADE levels. Both *JAMA* and GQS scores were higher in groups A and B compared with groups C and D, while group C consistently showed the lowest scores for all 3 metrics. Similarly, DISCERN scores were notably higher for groups A and B relative to groups C and D.

### Correlation Among Key Variables

**Figure 3.**
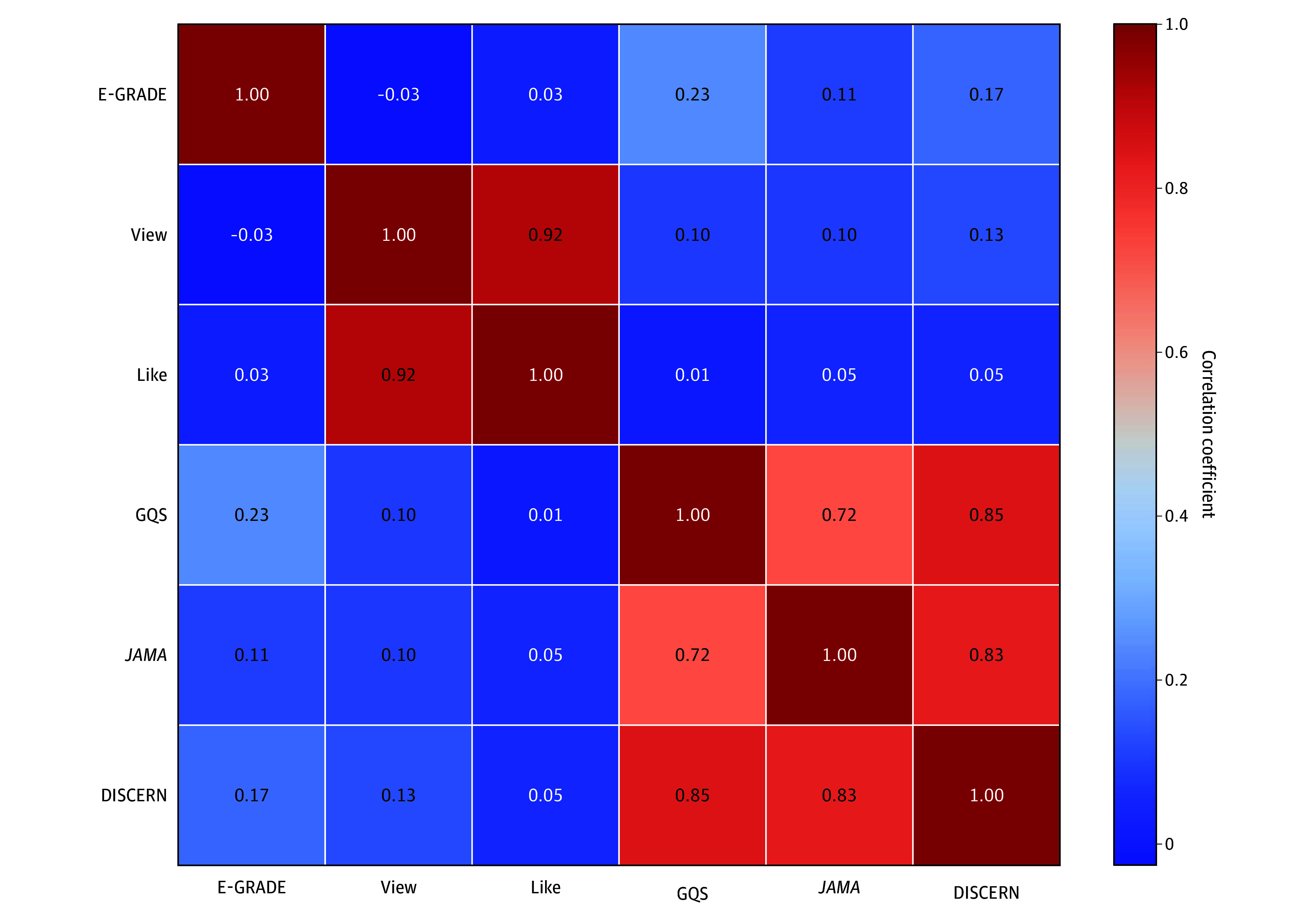
Spearman Correlation Matrix of Key Variables E-GRADE indicates Evidence-GRADE (Grading of Recommendations Assessment, Development and Evaluation); GQS, Global Quality Scale; *JAMA*, *JAMA* benchmark criteria.

Figure 3 shows the Spearman correlation matrix among major study variables, including E-GRADE, view count, like count, and GQS, *JAMA*, and DISCERN scores. E-GRADE exhibited weak correlations with other variables (range, –0.03 to 0.23). View and like counts were strongly correlated with each other (range of correlation coefficients, 0.80-1.00; *P* = .92) but displayed minimal correlations with quality scores or E-GRADE. In contrast, quality scores (GQS, *JAMA*, and DISCERN) were strongly interrelated, with correlation coefficients as high as 0.85 (GQS and DISCERN) and 0.83 (*JAMA* and DISCERN). However, traditional quality evaluation tools showed only weak correlations with evidence levels (range of correlation coefficients, 0.11-0.23). These results indicate that video popularity metrics are closely linked but not associated with evidence quality or overall quality scores.

### Association With Video View Counts and Likes

The results of the multivariate negative binomial regression, which adjusted for video topic, channel size, upload date, and video length, are presented in Table 2. After adjusting for covariates, claims with lower evidence levels showed higher view counts compared with grade A claims. This association was statistically significant for grade D videos, which were associated with a 34.6% higher view count ([IRR, 1.35; 95% CI, 1.00-1.81; *P* = .047) than grade A videos. While grade B (IRR, 1.41; 95% CI, 0.95-2.09; *P* = .09) and grade C (IRR, 1.90; 95% CI, 0.96-3.76]; *P* = .07) also showed higher point estimates for view counts, these differences did not reach statistical significance. Channel size (IRR, 1.16; 95% CI, 1.07-1.26), months since upload (IRR, 1.03; 95% CI, 1.02-1.04), and video length (IRR, 1.38; 95% CI, 1.20-1.59) were significant factors in view counts (*P* < .001 for all). A separate model analyzing engagement efficiency (likes per 1000 views) found no association between E-GRADE levels and this metric.

**Table 2.** Negative Binomial Regression Results for Video View and Like Counts^a^

Variable	IRR (95% CI)	*P* value
**View counts**		
E-GRADE		
Grades B vs A	1.41 (0.95-2.09)	.09
Grades C vs A	1.90 (0.96-3.76)	.07
Grades D vs A	1.35 (1.00-1.81)	.047
Covariates		
log(Channel size)	1.16 (1.07-1.26)	<.001
Months since upload	1.03 (1.02-1.04)	<.001
log(Length, s)	1.39 (1.20-1.59)	<.001
**Likes per view**		
E-GRADE		
Grades B vs A	1.03 (0.70-1.52)	.89
Grades C vs A	1.04 (0.53-2.06)	.90
Grades D vs A	0.89 (0.67-1.20)	.45
Covariates		
log(Channel size)	0.97 (0.90-1.06)	.52
Months since upload	0.99 (0.98-0.99)	<.001
log(Length, s)	1.06 (0.92-1.22)	.40

Abbreviations: E-GRADE, Evidence-GRADE (Grading of Recommendations Assessment, Development and Evaluation); IRR, incidence rate ratio.

^a^ Uses negative binomial regression model. Models adjust for topic (disease) fixed effects, log(channel size), months since upload, and log(length [in seconds]). The likes-per-view model uses an offset log(view) to estimate a rate.

### Post Hoc Analysis of Engagement Metrics by E-GRADE Level

eFigure 4 in Supplement 1 displays the post hoc comparison of video view and like counts across different E-GRADE levels. Both metrics displayed similar distributions across the 4 groups. No significant differences were found in view counts or like counts across E-GRADE levels.

## Discussion

In this quality improvement study of health care professional–generated online videos, we implemented the E-GRADE framework as a novel systematic approach to assessing the evidence hierarchy supporting medical video claims. Our findings revealed a large credibility-evidence gap: while 62.5% of medical claims were supported by very low or no evidence (grade D), only 19.7% were backed by high-quality evidence (grade A). Critically, traditional quality metrics (DISCERN, *JAMA*, and GQS) failed to capture this disparity, and importantly, there was no correlation between the strength of supporting evidence and engagement metrics such as view counts or likes.

This credibility-evidence gap represented what we term the halo effect of medical specialists’ authority, where medical credentials lend scientific legitimacy to claims lacking robust empirical support. While this professional halo effect is our focus, we recognize that in the current digital ecosystem, influence is not limited to credentialed experts. Nonmedical influencers often build trust through perceived authenticity and relatability. The professional halo effect, however, functions differently: it confers a veneer of scientific legitimacy derived from institutional authority, making health claims seem more objective and less questionable to the public. This distinction is critical because it highlights a unique challenge to evidence-based principles. The Federation of State Medical Boards has recognized that physician-spread misinformation poses particularly potent dangers because medical credentials give claims disproportionate weight in public discourse.^25^ Recent analysis of medical board disciplinary actions^26^ showed that despite concerns about physician misinformation, only 0.1% of proceedings involved spreading misinformation to the community. Medical boards cited misinformation to patients as grounds for discipline 3 times more often than for similar actions involving the general public.^26^

While our unadjusted analysis showed no statistically significant difference in raw view counts, an adjusted negative binomial regression model revealed a different story: grade D videos were associated with higher engagement after controlling for powerful confounders like video age. This finding highlights the importance of using adjusted models to isolate the true relationship between variables. This underlying inverse association reflects the study platform’s algorithm-driven environment that prioritizes viewer engagement over scientific rigor. The study platform’s algorithms can inadvertently promote emotionally charged or entertainment-focused content over serious, evidence-based information, creating systematic bias toward sensationalized content.^27,28^ This algorithmic preference for engagement-driven content undermines evidence-based medicine principles and creates an “epistemic crisis” wherein popularity becomes a proxy for scientific validity.^29,30,31^

It is crucial, however, to contextualize these findings within the broader media landscape. This study does not suggest that the gap between medical authority and empirical evidence is exclusive to social media. Scholarship has long critiqued the quality of health information and the potential for bias in traditional media, such as television and print news, where claims are not subject to peer review. The study platform, however, introduces unique mechanisms that may exacerbate this problem. Unlike traditional media, which retains some (albeit imperfect) editorial gatekeeping, the platform allows for direct, unmediated publishing. This structure, combined with an engagement-driven algorithm that can prioritize sensationalism over scientific rigor, creates a fertile ground for the credibility-evidence gap to flourish at an unprecedented scale and speed.

The E-GRADE framework addresses critical limitations in existing quality assessment tools. While DISCERN, *JAMA* criteria, and GQS evaluate presentation quality and source credibility, they cannot distinguish between well-presented videos containing anecdotal claims and those grounded in systematic reviews. The recently developed Principles for Health-Related Information on Social Media (PRHISM) framework for social media health information similarly emphasizes transparency and authorship but lacks systematic evidence hierarchy assessment.^32^ For this reason, while PRHISM represents an important advancement, it was not selected as a primary comparator tool, as its conceptual scope would not have addressed the specific evidence-based gap our study sought to investigate. Our approach helps identify the phenomenon where physician authority conceals insufficient evidence support.

Our findings align with emerging concerns about medical misinformation disseminated by licensed medical specialists.^33,34^ Professional medical organizations have increasingly recognized that physicians’ specialized knowledge and training creates a powerful platform in society with corresponding responsibility for factual, evidence-based information sharing.^35^ The American Academy of Family Physicians emphasizes that physicians using social media must behave professionally and avoid offering medical advice without a proper evidence foundation.^36^ However, current professional guidelines focus primarily on patient privacy and professional boundaries rather than evidence quality standards.

The predominance of low-evidence claims from credentialed sources suggests urgent needs for enhanced education in evidence-based communication and science literacy across all health disciplines. Our study included a range of licensed professionals, and this finding highlights that professional schools (including medical, pharmacy, nursing, and traditional medicine schools) and postgraduate training programs should integrate training on evidence hierarchy assessment, science communication principles, and the ethical responsibilities of public health communication. Furthermore, all professional health organizations need to create guidelines for their member content creators that are akin to clinical practice guidelines but tailored to public communication standards. While professional organizations such as the American Medical Association have established valuable social media guidelines, these often focus on professionalism and patient privacy rather than the quality of evidence underlying public-facing claims. Our findings suggest a critical need to enhance these existing guidelines by incorporating specific standards for evidence-based communication, ensuring that the authority of a professional license is matched by the rigor of the information shared.

### Limitations

This study has several limitations. First, the analysis was restricted to Korean- and English-language videos through Korean search terms addressing only cancer and diabetes, potentially limiting generalizability across different health care systems, cultural contexts, and medical conditions. A primary limitation is the survivorship bias introduced by our inclusion criterion of a minimum of 10 000 views. Although this threshold was established to focus on content with a significant public health reach, it necessarily excluded the large volume of videos that did not achieve this level of engagement. Second, we did not assess the potential educational benefits of accessible physician-generated content for health literacy improvement and patient engagement, focusing exclusively on evidence quality rather than broader educational outcomes. Third, our analysis did not account for potential clustering effects from videos by the same creator. Creator-level clustering was minimal in our sample (106 unique creators for 309 videos), but we acknowledge that this is an important consideration. Fourth, a key limitation is the inherent subjectivity within the E-GRADE criteria, such as the operational distinction between major and other organizations, or high-quality and limited studies. Finally, our E-GRADE framework was designed to objectively score the highest level of scientific evidence supporting a specific claim, not the appropriateness or responsibility of its presentation.

## Conclusions

This quality improvement study found that most medical claims made on the study platform had low-quality evidence. This reveals a substantial credibility-evidence gap in medical content videos, where physician authority frequently legitimizes claims lacking robust empirical support. The E-GRADE framework provides a novel methodological tool for evaluating evidence quality beyond traditional assessment measures, enabling systematic identification of this critical gap. Our findings underscore the necessity for evidence-based content creation guidelines, enhanced science communication training for health care professionals, and algorithmic reforms prioritizing scientific rigor alongside engagement metrics. As social media platforms increasingly shape public health discourse, ensuring alignment between expert authority and evidence-based medicine represents a fundamental challenge for maintaining scientific integrity in the digital age. The proliferation of physician-generated content lacking evidence standards threatens both individual patient care and broader public health outcomes, necessitating intervention from medical education institutions, professional organizations, and regulatory bodies.

## Notes

Supplement 1. **eTable.** Comparison of Quality Scores (GQS, *JAMA*, and DISCERN) according to E-GRADE Levels **eFigure 1.** The Systematic Process of Video Identification, Screening, and Inclusion **eFigure 2.** Distribution of Evidence Levels (E-GRADE) for Claims in Videos **eFigure 3.** Engagement Efficiency by E-GRADE Level **eFigure 4.** Post Hoc Analysis of Engagement Metrics by E-GRADE Level **eAppendix 1.** Search Strategy and Terms **eAppendix 2.** Example of a Coding Sheet

Supplement 2.
Data Sharing Statement
