# Supplementary material for: The Quality of Evidence of and Engagement With Video Medical Claims
Source: JAMA Netw Open. 2026 Jan 16;9(1):e2552106. doi: 10.1001/jamanetworkopen.2025.52106 (PMC12811808; doi:10.1001/jamanetworkopen.2025.52106)
Supplement: Supplement 1. — eTable. Comparison of Quality Scores (GQS, JAMA, and DISCERN) according to E-GRADE Levels eFigure 1. The Systematic Process of Video Identification, Screening, and Inclusion eFigure 2. Distribution of Evidence Levels (E-GRADE) for Claims in Videos eFigure 3. Engagement Efficiency by E-GRADE Level eFigure 4. Post Hoc Analysis of Engagement Metrics by E-GRADE Level eAppendix 1. Search Strategy and Terms eAppendix 2. Example of a Coding Sheet [file jamanetwopen-e2552106-s001.pdf]

Supplementary Online Content

Kang E, Lee H, Choi J, Ju H. The quality of evidence of and engagement with video medical claims. *JAMA Netw Open.* 2026;9(1):e2552106.
doi:10.1001/jamanetworkopen.2025.52106

eTable. Comparison of Quality Scores (GQS, *JAMA*, and DISCERN) According to E-GRADE Levels

eFigure 1. The Systematic Process of Video Identification, Screening, and Inclusion

eFigure 2. Distribution of Evidence Levels (E-GRADE) for Claims in Videos

eFigure 3. Engagement Efficiency by E-GRADE Level

eFigure 4. Post Hoc Analysis of Engagement Metrics by E-GRADE Level

eAppendix 1. Search Strategy and Terms

eAppendix 2. Example of a Coding Sheet

This supplementary material has been provided by the authors to give readers additional information about their work.

© 2026 Kang E et al. *JAMA Network Open*.

eTable. Comparison of Quality Scores (GQS, *JAMA*, and DISCERN) According to E-GRADE Levels

	N	GQS (Mean ± SD)	*JAMA* (Mean ± SD)	DISCERN (Mean ± SD)
**A**	61	3.51 ± 0.77	2.64 ± 1.02	55.44 ± 11.65
**B**	45	3.62 ± 1.01	2.69 ± 1.02	56.16 ± 13.93
**C**	10	2.90 ± 0.74	2.20 ± 1.14	45.10 ± 15.25
**D**	193	3.06 ± 0.88	2.40 ± 1.02	49.50 ± 14.91
**p-value[1]**		< 0.001	0.152	0.003

Footnote: *Scores represent video-level data, where each video was assigned a primary E-GRADE based on its main health claim. Bold values indicate statistical significance (P < .05).* [1] *Overall p-values were determined by the Kruskal-Wallis test. For significant results, post-hoc Conover's tests with Bonferroni correction were performed. Significant pairwise adjusted p-values were as follows:*

- ***GQS Scores:*** *Group A vs D (p = .005); Group B vs D (p = .001).*
- ***DISCERN Scores:*** *Group B vs D (p = .019).*

© 2026 Kang E et al. *JAMA Network Open*.

eFigure 1. The Systematic Process of Video Identification, Screening, and Inclusion

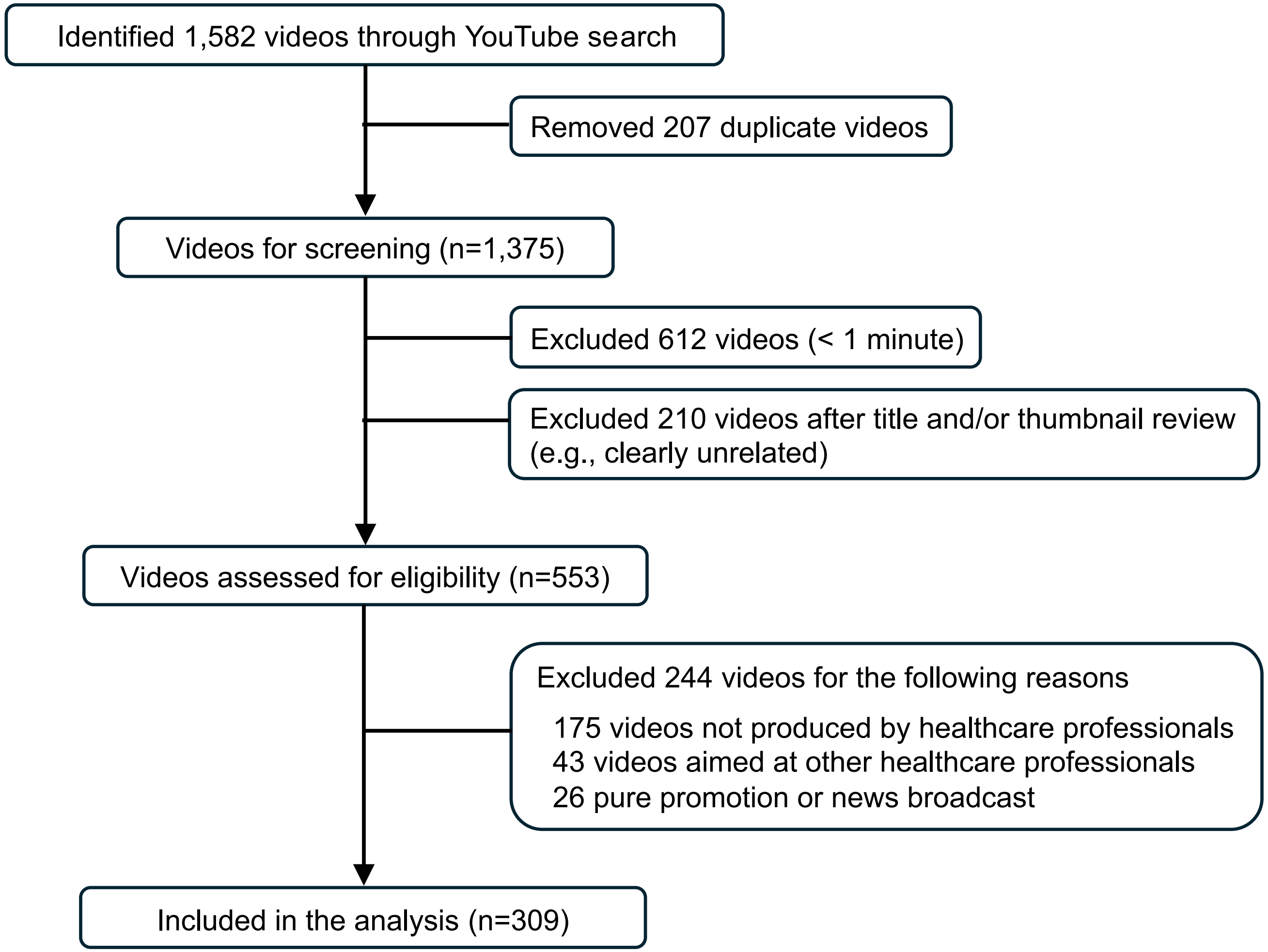

© 2026 Kang E et al. *JAMA Network Open*.

eFigure 2. Distribution of Evidence Levels (E-GRADE) for Claims in Videos

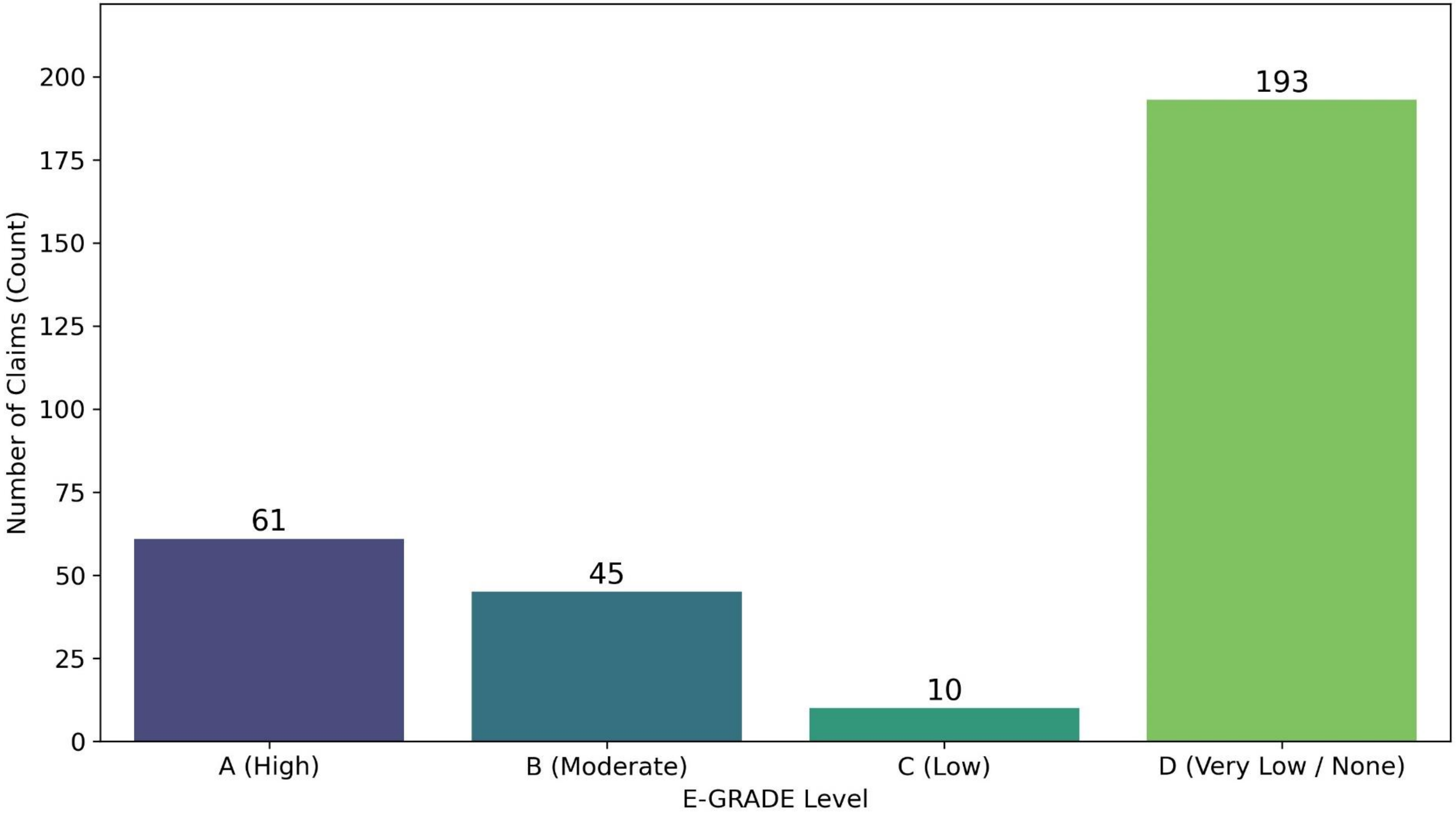

© 2026 Kang E et al. *JAMA Network Open*.

eFigure 3. Engagement Efficiency by E-GRADE Level

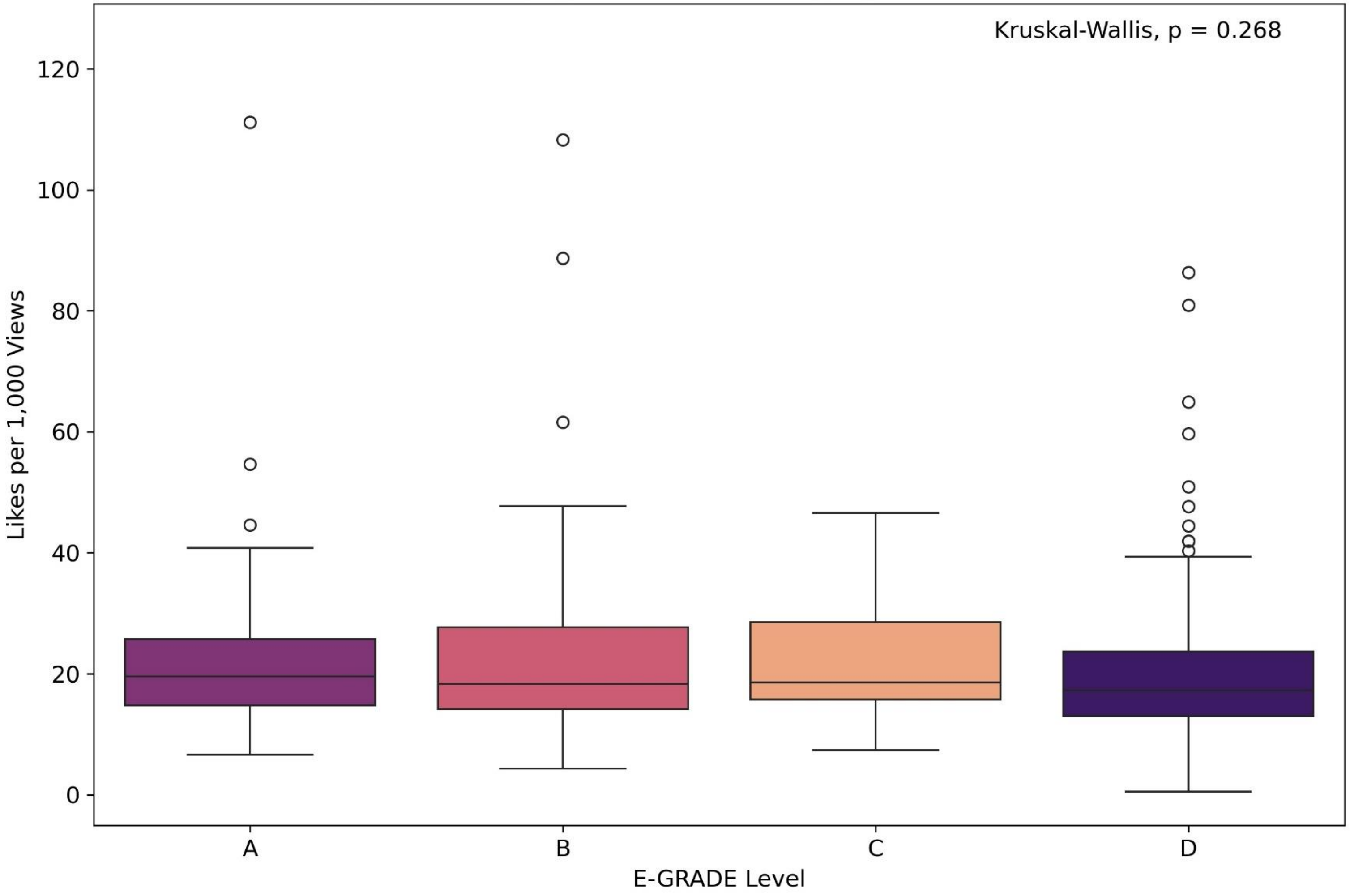

© 2026 Kang E et al. *JAMA Network Open*.

eFigure 4. Post Hoc Analysis of Engagement Metrics by E-GRADE Level

(**eFigure 4A.** View Count by E-GRADE Level (Left); **eFigure 4B.** Like Count by E-GRADE Level (Right))

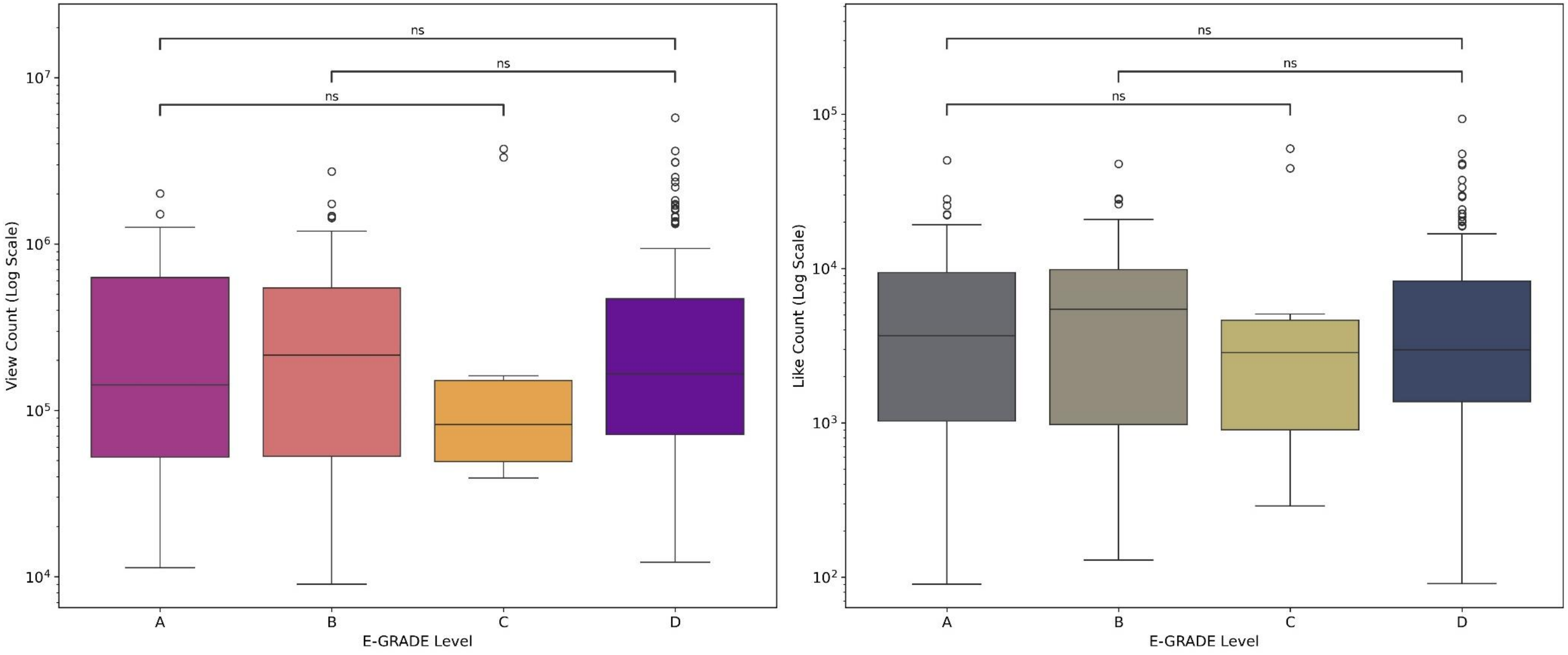

© 2026 Kang E et al. *JAMA Network Open*.

eAppendix 1. Search Strategy and Terms

Introduction

To identify a comprehensive and representative sample of videos for this study, a systematic search was conducted on YouTube using both English and Korean search terms. The search strategy was designed to mirror the diverse ways in which the public seeks health information online. This included using a combination of formal medical terminology (e.g., "chemotherapy") and common lay terminology (e.g., "foods that fight cancer"). The full list of search terms is detailed below.

Cancer-Related Search Terms

Our search for cancer-related content began with broad, foundational terms. In English, these included "Cancer," "Cancer treatment," "Cancer prevention," "Cancer symptoms," and "Cancer diet." The corresponding Korean terms used were "Am", "Am chiryo", "Am yebang", "Am jeungsang", and "Am sikdan". We also included searches for specific treatment modalities such as "Chemotherapy" ("Hang-am chiryo"), "Immunotherapy" ("Myeon-yeok hang-amje"), and "Radiation therapy" ("Bangsaseon chiryo").

To ensure our search captured content on the most relevant and frequently discussed cancer types in the South Korean context, we included terms for cancers with the highest incidence rates in the country. These specific cancer search terms were: "Stomach cancer" ("Wiam"), "Colon cancer" ("Daejang-am"), "Thyroid cancer" ("Gapsangseon-am"), "Lung cancer" ("Pye-am"), "Breast cancer" ("Yubang-am"), "Prostate cancer" ("Jeollipseon-am"), and "Cervical cancer" ("Jagung-gyeongbu-am").

Finally, to include content related to alternative or lifestyle-based approaches, which are prevalent on the platform, we searched for terms like "Natural cancer cure" ("Am jayeon chiyu"), "Foods that fight cancer" ("Am igineun eumsik"), and "Foods good for cancer" ("Ame jo-eun eumsik").

Diabetes-Related Search Terms

For diabetes-related content, our search followed a similar strategy. Broad English terms included

© 2026 Kang E et al. *JAMA Network Open*.

“Diabetes,” “Type 2 diabetes,” “Type 1 diabetes,” and “Prediabetes.” The Korean equivalents were “Dangnyobyeong” or “Dangnyo”, “Ihyeong dangnyo”, “Ilhyeong dangnyo”, and “Dangnyo jeondangye”. We also searched for common patient queries, such as “How to lower blood sugar” (“Hyeoldang nat-chuneun beop”), “High blood sugar” (“Gohyeoldang”), and “Reversing diabetes” (“Dangnyo wanchi”).

Searches related to management and treatment included “Diabetes treatment” (“Dangnyo chiryo”), “Diabetes diet” (“Dangnyo sikdan”), and terms for common medications like “Metformin” (“Meteuporeumin”) and “Insulin” (“Insullin”). We also included “Gestational diabetes” (“Imsinseong dangnyo”) due to its specific information needs. To capture dietary advice, we used terms like “Foods good for diabetes” (“Dangnyo-e jo-eun eumsik”) and “Foods to avoid with diabetes” (“Dangnyo-e nappeun eumsik”).

© 2026 Kang E et al. *JAMA Network Open*.

eAppendix 2. Example of a Coding Sheet

	Variable	Data
Section A: Basic Video Information	Study Video ID:	
	Video Title:	
	Video URL:	
	Date of Extraction:	YYYY-MM-DD
	Reviewer Initials:	
Section B: Creator & Video Characteristics	Creator's Profession:	□ Medical Doctor □ Dentist □ Traditional Korean Medicine Doctor □ Pharmacist □ Nurse □ Herbal Pharmacist □ Other (Specify):
	Primary Topic:	□ Cancer □ Diabetes
	Date of Upload:	YYYY-MM-DD
	Video Length (minutes):	
Section C: Engagement Metrics (at time of extraction)	View Count:	
	Like Count:	
	Comment Count:	
Section D: E-GRADE Assessment of the Main Medical Claim	Assign E-GRADE (Select one):	□ Grade A (High certainty): Supported by systematic reviews/meta-analyses or major clinical guidelines.<br>□ Grade B (Moderate certainty): Supported by RCTs or high-quality observational studies.<br>□ Grade C (Low certainty): Supported by limited observational studies, physiological rationale, or case series.<br>□ Grade D (Very low/No certainty): Based on anecdote, personal experience, or unsupported assertions.

© 2026 Kang E et al. *JAMA Network Open*.

	Justification for E-GRADE:	
Section E: Traditional Quality Assessment Scores	1. DISCERN Score (Total):	________ / 80
	1-1. Section 1: Reliability	________ / 40
	1-2. Section 2: Quality of Treatment Info	________ / 35
	1-3. Overall Quality Rating	________ / 5
	2. JAMA Benchmark Criteria (Total):	________ / 4
	2-1. Authorship	________ / 1
	2-2. Attribution	________ / 1
	2-3. Disclosure	________ / 1
	2-4. Currency	________ / 1
	3. Global Quality Scale (GQS) Score:	________ / 5
Section F: Additional Reviewer Notes		

© 2026 Kang E et al. *JAMA Network Open*.
