# Supplementary material for: The Quality of Evidence of and Engagement With Video Medical Claims
Source: JAMA Netw Open. 2026 Jan 16;9(1):e2552106. doi: 10.1001/jamanetworkopen.2025.52106 (PMC12811808; doi:10.1001/jamanetworkopen.2025.52106)
Supplement: Supplement 2. — Data Sharing Statement [file jamanetwopen-e2552106-s002.pdf]

Data Sharing Statement

Kang. The Quality of Evidence of and Engagement With Video Medical Claims. *JAMA Netw Open*. Published January 16, 2026. doi:10.1001/jamanetworkopen.2025.52106

Data
Data available: Yes
Data types: Data (not involving human participants)
How to access data: The data that support the findings of this study are available from the corresponding author (ekherb@ncc.re.kr) upon reasonable request for research purposes.
When available: With publication

Supporting Documents
Document types: None

Additional Information
Who can access the data: researchers whose proposed use of the data has been approved
Types of analyses: reasonable request for research purposes
Mechanisms of data availability: after approval of a proposal
